# Red blood cell count and its inverse association with diabetic retinopathy: Exploratory development of a risk assessment model in a retrospective cohort

**DOI:** 10.3389/fendo.2025.1571192

**Published:** 2025-08-08

**Authors:** Jing Li, Qian Xu, Xiao Yuan, Wen Hu

**Affiliations:** ^1^ Department of Endocrinology and Metabolism, Suqian First Hospital, Suqian, Jiangsu, China; ^2^ Department of Endocrinology and Metabolism, Huai’an Hospital Affiliated to Xuzhou Medical University and Huai’an Second People’s Hospital, Huai’an, Jiangsu, China

**Keywords:** red blood cell count, diabetic retinopathy, risk factor, model, prediction, nomogram

## Abstract

**Objective:**

The purpose of this exploratory study was to investigate the association between red blood cell count (RBC) and diabetic retinopathy (DR) and to develop a preliminary risk assessment framework.

**Methods:**

A total of 413 individuals diagnosed with type 2 diabetes mellitus (T2DM) at Suqian First Hospital’s Endocrinology Department were included in this study. These participants were divided into training and validation groups in a 7:3 ratio, consisting of 289 and 124 patients respectively. In the training cohort, potential predictive variables were determined through both univariate and multivariate analyses utilizing forward-backward stepwise selection. Only variables with p < 0.05 were included in the nomogram, which encompassed demographic information, clinical laboratory results, and diabetes-associated complications. The performance of the model was evaluated in both groups using receiver operating characteristic (ROC) curve analysis, the Hosmer-Lemeshow test for calibration, and decision curve analysis (DCA) to determine clinical utility.

**Results:**

Out of 20 clinical variables examined, five were chosen to develop the nomogram: RBC, serum creatinine (SCR), diabetes duration, diabetic peripheral neuropathy (DPN), and diabetic kidney disease (DKD). The ROC analysis revealed that the area under the curve (AUC) for the training cohort was 0.765 (95% CI 0.709-0.821) and for the validation cohort was 0.707 (95% CI 0.616-0.798). Results from the Hosmer-Lemeshow test were p = 0.233 and p = 0.579, indicating a good fit. The nomogram demonstrated excellent predictive accuracy and provides a quantitative tool for assessing the risk of DR in individuals with T2DM.

**Conclusion:**

Our findings suggest an inverse association between RBC levels and DR risk. The exploratory model incorporating RBC provides an initial framework for evaluating DR risk in patients with T2DM. Further validation in prospective cohorts is needed to refine this framework before considering clinical applications.

## Introduction

T2DM, the most prevalent form of diabetes, is primarily associated with insulin resistance (IR) and metabolic syndrome (MS). These conditions contribute to the progressive non-immune damage of pancreatic β-cells and decreased insulin secretion (1). Over the last century, the incidence of T2DM has escalated significantly, raising concerns within the global health community (2, 3). Although the incidence stabilized or declined slightly after the mid-2000s, the overall number of individuals affected by diabetes remains high (2). DR, a microvascular complication of diabetes, leads to vision impairment and blindness in T2DM patients by damaging the blood vessels in the retina (4, 5). With an aging population and longer durations of diabetes, the prevalence of DR increased from 14.9% in 1990 to 18.5% in 2020 (5, 6). DR increases the risk of vision loss through retinal ischemia, hemorrhage, and exudation (7). Although management strategies such as controlling blood glucose and blood pressure can slow DR progression, and treatments like laser photocoagulation and intravitreal injections of anti-vascular endothelial growth factor (anti-VEGF) are available, these methods face significant limitations and challenges (7).

The development and progression of DR are influenced by various factors including diabetes duration, control, blood pressure, and lipid profiles (7). Recent studies have highlighted the role of hematological factors like platelet count and RBC, in the pathogenesis of DR (8, 9). While traditional risk factors such as chronic hyperglycemia and hypertension are well-established, the potential impact of RBC on DR is less explored. RBCs are essential for oxygen transport and tissue oxygenation, and their variations might affect retinal blood flow and microvascular health, thereby influencing DR severity (9). An increase in RBC count has been linked to higher blood viscosity, which may impair microcirculatory function and worsen damage to retinal vessels (9). Conversely, a reduced RBC count might indicate anemia, potentially harming retinal health. Therefore, elucidating the relationship between RBC and DR could offer new insights into the disease mechanisms and enhance risk assessment and management strategies (10).

Risk prediction models that utilize various clinical parameters to estimate individual disease risk have become valuable tools alongside traditional screening methods. By integrating robust risk prediction models, screening practices can be enhanced, allowing for more effective DR prevention and personalized management. Although several predictive models have been employed for DR recognition and diagnosis, the correlation between RBC and DR is often overlooked in these models (11–18). Thus, this study aims to employ logistic regression analysis to investigate the correlation between RBC and DR, explore the role of RBC and other clinical parameters in early DR screening and prediction, and develop a risk nomogram for DR prediction.

## Methods

### Study population

**Figure 1:**
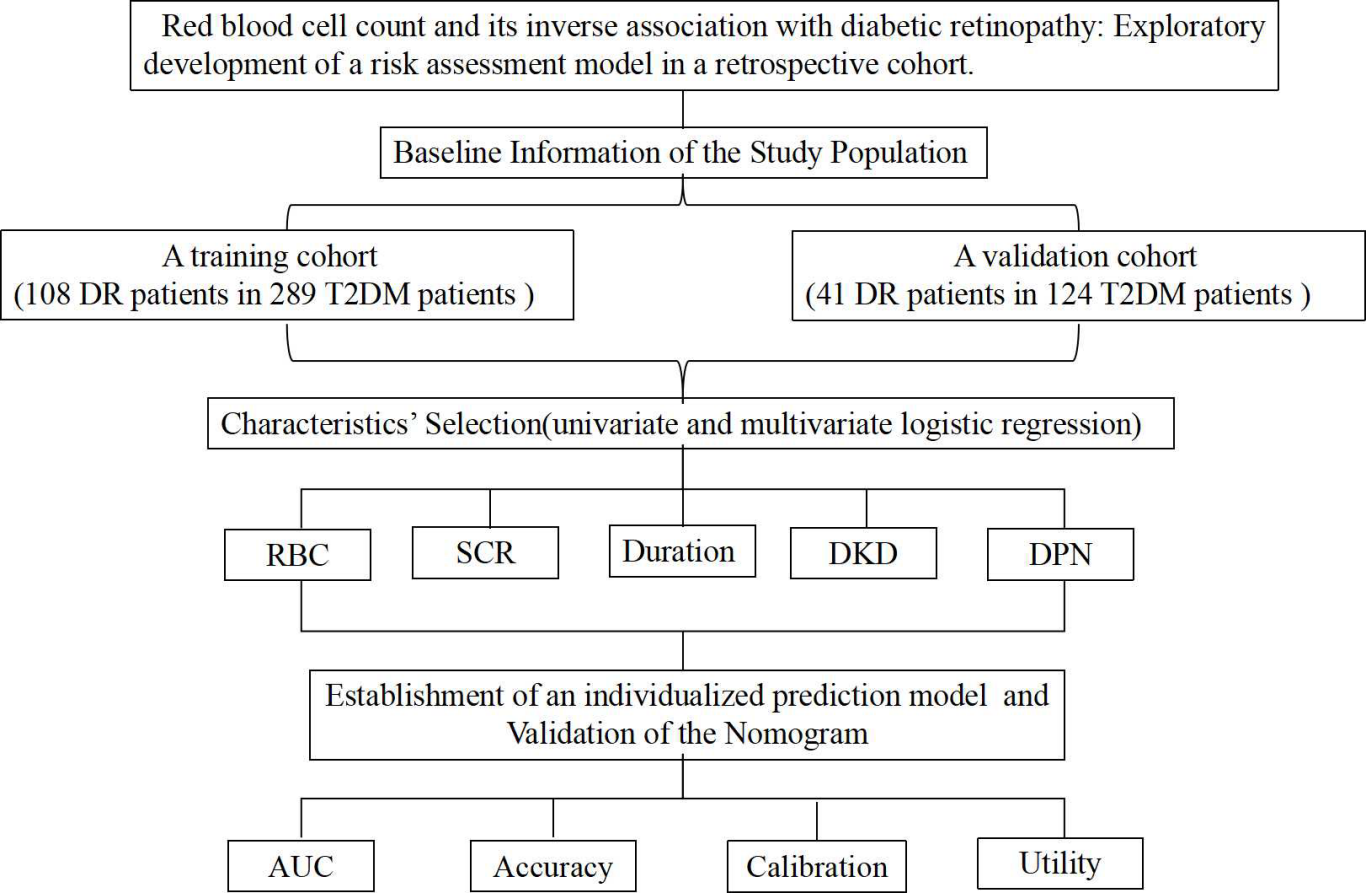
Flow chart of our study.

Between January 2021 and December 2023, 413 patients hospitalized with T2DM at Suqian First Hospital’s Department of Endocrinology were included. **Figure 1** shows the study’s flowchart. Suqian First Hospital’s Ethics Committee gave their stamp of approval with the following number: 2024-SL-0136.

### Inclusion and exclusion criteria

#### Inclusion criteria

Individuals had to be 18 or older and have a diagnosis of T2DM as per the American Diabetes Association’s criteria (1). They were then evaluated for diabetic complications, such as DR, diabetic peripheral vascular disease (DPVD), and DKD.

#### Exclusion criteria

Excluded were participants without a diabetes diagnosis; those with acute illnesses, including infections or inflammatory conditions; a history of hematological disorders such as anemia or polycythemia; any surgical procedure or significant trauma within the past three months; pregnant or breastfeeding women; those with significant cardiovascular, renal, or hepatic diseases; and those on medications affecting RBC counts or retinal health, such as corticosteroids or certain chemotherapeutics.

### Diagnostic criteria

#### DR

Diagnosis was based on fundus photographic examination revealing microaneurysms, retinal hemorrhage, exudation, and neovascularization.

#### DPVD

Diagnosed through clinical assessments and ancillary tests including patient history, physical examinations of pulse presence and skin characteristics, and diagnostic tests such as the ankle-brachial index (ABI) and arteriography.

#### DKD

Diagnosis was confirmed by clinical evaluation and laboratory tests, including persistent albuminuria and a decline in glomerular filtration rate (GFR).

#### DPN

These criteria must be met in order to make a diagnosis of diabetic neuropathy: a confirmed diagnosis of diabetes or abnormal glucose metabolism; the onset of neuropathy either at the same time as or after the diabetes diagnosis; clear electrophysiological or clinical evidence of nerve damage associated with diabetes; and other possible causes of peripheral neuropathy must be ruled out.

### Clinical data collection

Clinical indicators collected included: sex, age, duration of diabetes, body mass index (BMI), white blood cell (WBC), red blood cell (RBC), platelet (PLT), glycated hemoglobin A1c (HbA1c), total cholesterol (TC), triglycerides (TG), high-density lipoprotein cholesterol (HDL-C), low-density lipoprotein cholesterol (LDL-C), aspartate aminotransferase (AST), alanine aminotransferase (ALT), SCR, albumin (ALB), fasting plasma glucose (FPG), and complications.

### Training and validation of the nomogram

The participants were divided into two groups: one for training (289 individuals) and another for validation (124 subjects), with a ratio of 7:3. Making receiver operating characteristic (ROC) curves and then determining the AUC allowed us to assess the model’s performance. In order to ensure that the model was properly calibrated, we utilized calibration curves to compare the expected outcomes with the actual clinical data, and we employed the Hosmer-Lemeshow (HL) test to confirm this. To determine if the model was useful in clinical settings, DCA was used.

### Statistical analysis

Statistical analysis was carried out using R version 4.2.1. Data are presented as mean ± standard deviation (SD). Comparison between groups was performed using Student’s t-test for normally distributed continuous variables, the Wilcoxon rank-sum test for non-normally distributed continuous variables, and the chi-squared test for categorical variables. To identify significant predictors, univariate and multivariate analyses were conducted, with variables showing p ≤ 0.05 included in a nomogram developed using a forward-backward stepwise method. The ‘rms’ package facilitated the creation of the nomogram and calibration curve, and the model’s predictive performance was evaluated.

## Results

### Characteristics of study

A total of 413 patients were enrolled, with 289 in the training cohort and 124 in the validation cohort (**Figure 1**, **Table 1**). In the training cohort, 108 patients developed DR, resulting in a prevalence of 37.4%, while 41 patients in the validation cohort had a prevalence of 33.1%. All variables analyzed, except for ALT, showed no statistically significant differences (P<0.05), confirming their comparability.

**Table 1:** Baseline characteristics of patients in the training cohort and validation cohort.

Variables	Total (n = 413)	Training cohort (n =289)	Validation cohort (n =124)	P value
Sex, n,%				0.298
Male	155 (3750)	106 (36.70)	49 (39.50)	
Female	258 (62.50)	183 (63.30)	75 (60.50)	
Age (years)	55.61 ± 13.91	55.26 ± 14.32	56.44 ± 12.94	0.333
Duration (years)	9.82 ± 7.67	9.63 ± 7.62	10.27 ± 7.78	0.853
BMI (kg/m^2^)	25.31 ± 3.94	25.37 ± 4.07	25.17 ± 3.65	0.230
WBC (×10^9/L)	6.19 ± 1.80	6.13 ± 1.74	6.33 ± 1.94	0.999
RBC (×10^12/L)	4.56 ± 0.62	4.58 ± 0.60	4.51 ± 0.68	0.385
PLT (×10^9/L)	208.04 ± 59.05	208.91 ± 60.07	206.02 ± 56.77	0.540
HbAlc (%)	9.45 ± 2.20	9.56 ± 2.18	9.20 ± 2.25	0.510
TC (mmol/L)	4.62 ± 1.22	4.63 ± 1.27	4.61 ± 1.09	0.573
TG (mmol/L)	1.33 (0.99-2.04)	1.33 (0.98-2.05)	1.35 (0.98-2.00)	0.430
HDL-C (mmol/L)	1.16 ± 0.32	1.16 ± 0.34	1.15 ± 0.26	0.198
LDL-C (mmol/L)	3.02 ± 0.87	3.01 ± 0.91	3.04 ± 0.79	0.593
AST (u/L)	21.37 ± 13.86	22.13 ± 15.64	19.59 ± 8.12	0.086
ALT (u/L)	18.80 (13.40-26.60)	19.80 (14.30-27.65)	17.55 (12.13-24.68)	0.023
SCR (umol/L)	56.05 ± 16.92	55.90 ± 16.32	56.43 ± 18.30	0.162
ALB (g/L)	39.85 ± 4.07	39.94 ± 4.10	39.63 ± 4.02	0.806
FPG (mmol/L)	8.06 ± 3.22	8.17 ± 3.12	7.82 ± 3.45	0.823
DPN, n (%)				0.865
No	164 (39.70)	119 (41.20)	45 (36.30)	
Yes	249 (60.30)	170 (58.80)	79 (63.70)	
DPVD, n (%)				0.155
No	139 (33.70)	99 (34.30)	40 (32.30)	
Yes	274 (66.30)	190 (65.70)	84 (67.70)	
DKD, n (%)				0.173
No	351 (85.00)	247 (85.50)	104 (83.90)	
Yes	62 (15.00)	42 (14.50)	20 (16.10)	
DR, n (%)				
No	264 (63.90)	181 (62.60)	83 (66.90)	0.698
Yes	149 (36.10)	108 (37.40)	41 (33.10)	

### Risk factors screening

Univariate logistic regression identified several significant predictors in the training cohort, including sex, age, duration of diabetes, BMI, RBC, SCR, ALB, DPN, DPVD, and DKD (P<0.10, **Table 2**). Multivariate regression further isolated independent risk factors for DR in patients with T2DM: duration of diabetes (OR = 1.04, 95% CI: 1.00 to 1.08), RBC(OR = 0.58, 95% CI: 0.36 to 0.94), SCR (OR = 0.98, 95% CI: 0.96 to 0.99), DPN (OR = 4.15, 95% CI:2.20 to 7.83) and DKD(OR = 3.49, 95% CI: 1.56 to 7.80) (**Table 2**).

**Table 2:** Univariate and multivariate logistic regression analyses for patients with T2DM.

Variables	OR (95%CI)	P value	OR (95%CI)	P value
Sex, n,%	0.63 (0.38-1.03)	0.063		
Age (years)	1.02 (1.00-1.04)	0.013		
Duration (years)	1.09 (1.05-1.13)	<0.001	1.04 (1.00-1.08)	0.040
BMI (kg/m^2^)	0.94 (0.88-1.00)	0.040		
WBC (×10^9/L)	1.01 (0.88-1.16)	0.909		
RBC (×10^12/L)	0.46 (0.30-071)	<0.001	0.58 (0.36-0.94)	0.028
PLT (×10^9/L)	1.00 (0.99-1.00)	0.198		
HbAlc (%)	1.09 (0.98-1.22)	0.128		
TC (mmol/L)	1.01 (0.83-1.21)	0.958		
TG (mmol/L)	0.99 (0.88-1.13)	0.929		
HDL-C (mmol/L)	1.74 (0.85-3.55)	0.126		
LDL-C (mmol/L)	0.82 (0.62-1.08)	0.161		
AST (u/L)	1.00 (0.98-1.01)	0.861		
ALT (u/L)	0.99 (0.98-1.01)	0.281		
SCR (umol/L)	0.99 (0.97-1.00)	0.086	0.98 (0.96-0.99)	0.006
ALB (g/L)	0.92 (0.86-0.98)	0.006		
FPG (mmol/L)	0.98 (0.91-1.06)	0.623		
DPN, n (%)	5.78 (3.25-10.29)	<0.001	4.15 (2.20-7.83)	<0.001
DPVD, n (%)	1.98 (1.17-3.36)	0.011		
DKD, n (%)	3.27 (1.66-6.43)	0.001	3.49 (1.56-7.80)	0.002

### Creation of the nomogram

**Figure 2:**
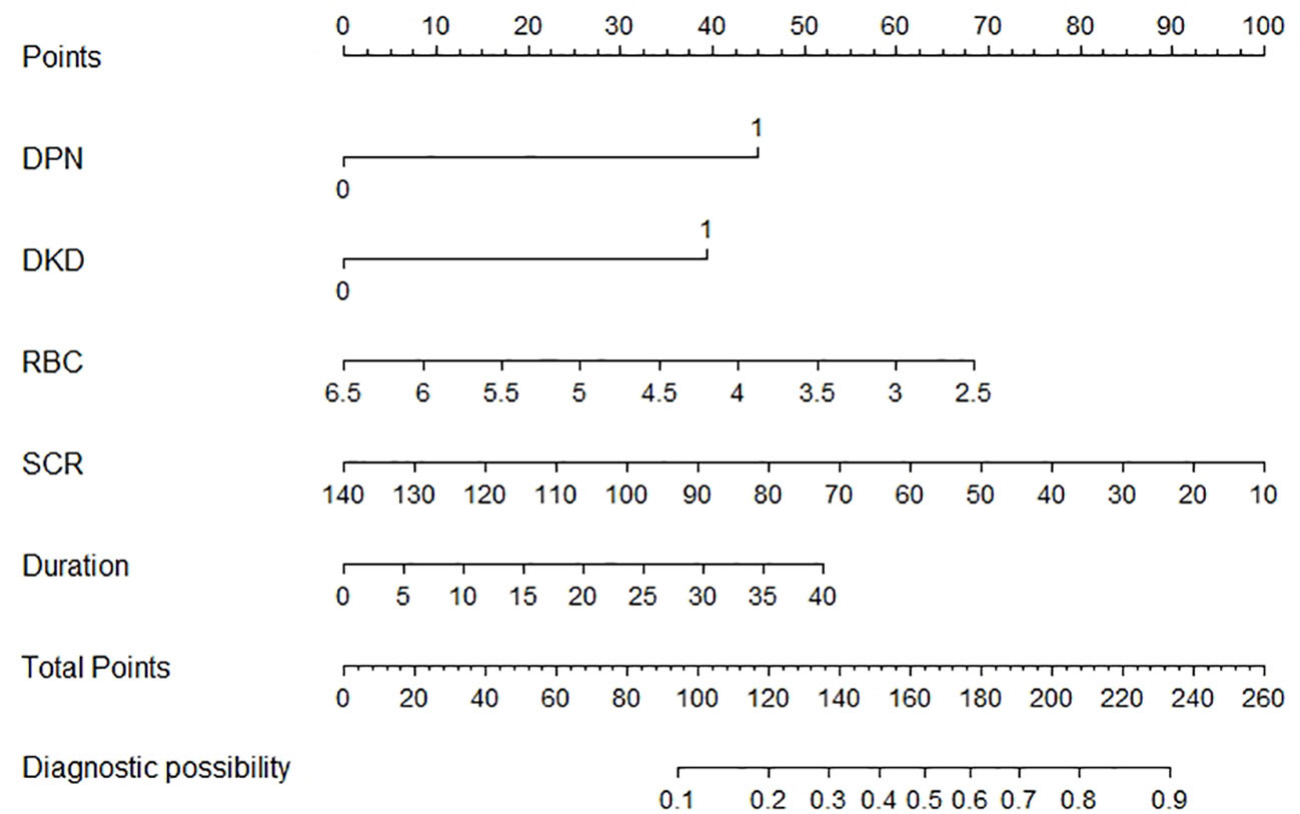
The nomogram model for quantifying individual risk of DR in patients with T2DM.

A nomogram incorporating these five risk factors was constructed (**Figure 2**). Points were assigned to each risk factor based on their contribution within the nomogram, and the sum of these points yielded an overall risk score, ranging from 0 to 260, corresponding to a predicted risk of developing DR from 0.1 to 0.9. A higher score indicated a higher risk of DR.

### Validation of the nomogram

**Figure 3:**
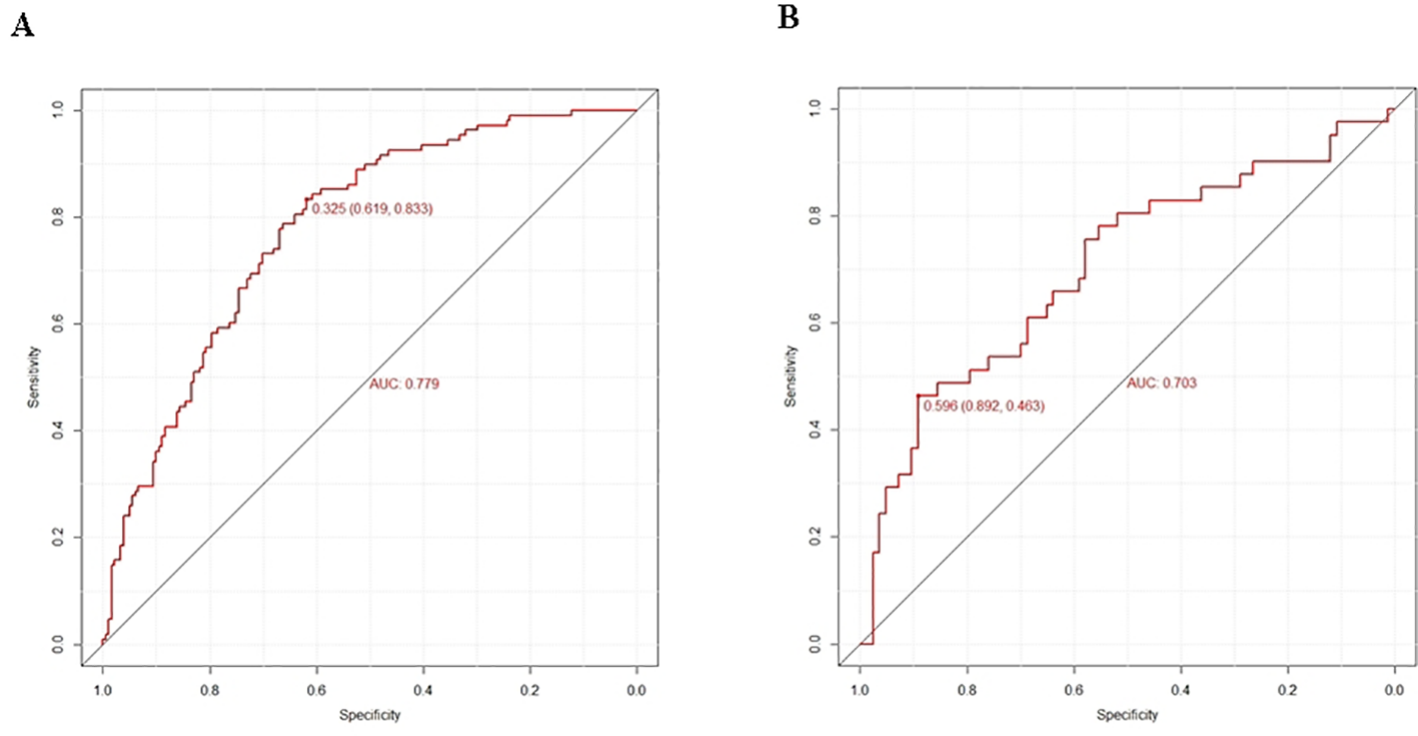
Prediction performance of the model. ROC curve plot in the training cohort **(A)** ROC curve plot in the validation cohort **(B)** AUC, the area under the ROC.

**Figure 4:**
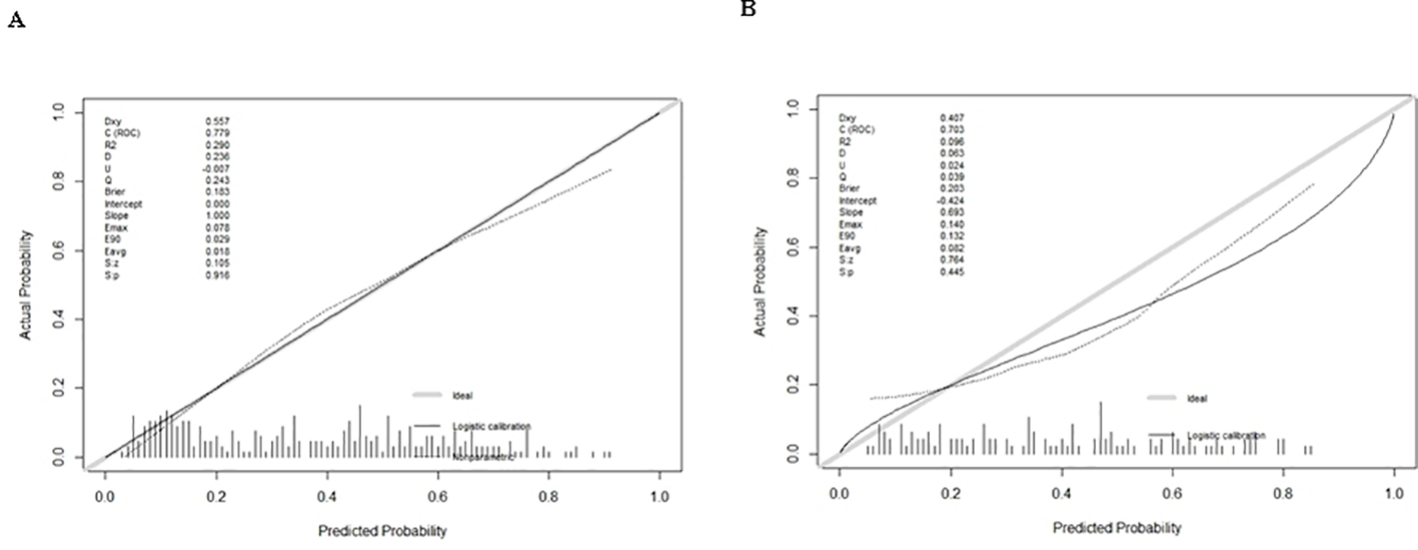
Calibration curve plot in each cohort. **(A)** the training cohort; **(B)** the validation cohort.

**Figure 5:**
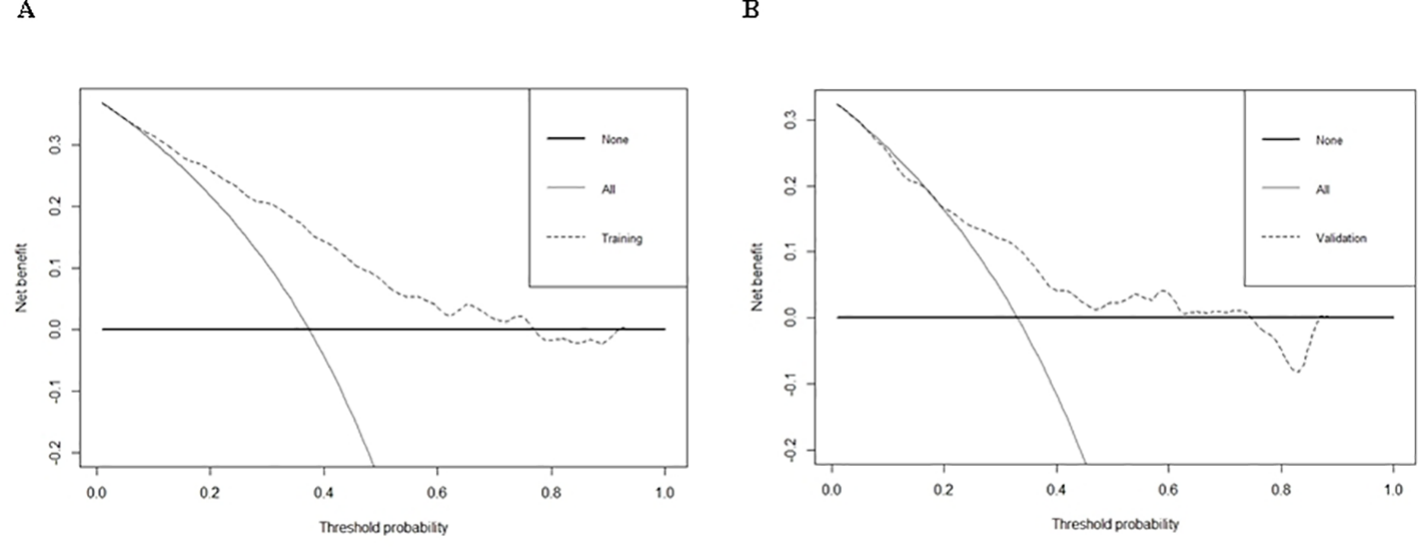
DCA of training cohort **(A)** and validation cohort **(B)** for the risk of DR in patients with T2DM.

Logistic regression provided predictive probabilities, with ROC analysis showing an AUC of 0.779 (95% CI: 0.726-0.832) for the development cohort and 0.703 (95% CI: 0.601-0.805) for the validation cohort, indicating good discrimination (**Figure 3**). Calibration curves (**Figure 4**) showed good agreement between predicted and actual outcomes, with HL tests yielding Chi-square values of 2.663 (p = 0.976) for the training cohort and 12.699 (p = 0.177) for the validation cohort. DCA in **Figure 5** demonstrated the nomogram’s clinical utility, with a net benefit at threshold probabilities of 10-75% and 20-75%. The nomogram outperformed no treatment and universal treatment scenarios within these thresholds.

## Discussion

In this study, DR occurred in more than one-third (up to 36.10%) of patients with T2DM, with a prevalence of 37.4% in the training cohort versus 33.1% in the validation cohort. DR is a primary cause of vision impairment and blindness among adults(4, 7). Its impact is significant, as individuals may incur increased medical costs for treatment and rehabilitation, alongside potential loss of income due to disability. Therefore, early detection of individuals at high risk is crucial to prevent DR and mitigate its detrimental effects on individuals and society, especially in the early stages of diabetes.

This exploratory analysis observed an inverse association between RBC count and DR risk in individuals with T2DM. The results indicate that RBC, in conjunction with DPN, DKD, SCR, and Duration, may be incorporated into a nomogram for predicting the risk of DR, thereby offering clinicians an essential instrument for the early detection and management of this complication. One patient was randomly selected from the population based on the characteristic indicators selected by the model. Patient indicators were as follows: DPN = yes, DKD = yes, RBC = 5×10^12/L, SCR = 100 umol/L, Duration = 10 years. We estimated individual DR Risk based on the total score (**Figure 2**). If the estimated risk of DR is greater than 50%, we should actively improve the fundus examination to screen for DR. If the risk is less than 10%, fundus examinations can be temporarily withheld to reduce medical expenses. While the model’s discrimination is moderate, its clinical utility lies in the inclusion of hematological parameters, which are routinely measured and cost-effective and machine learning approaches (e.g., ensemble models) may further enhance performance and will be explored in future work.

RBCs, or erythrocytes, are specialized cells responsible for transporting oxygen from the lungs to the body’s tissues and returning carbon dioxide for exhalation (9). The association of higher RBC counts with a reduced risk of DR is noteworthy and warrants further investigation. RBCs impact DR by altering membrane proteins, reducing surface charge, and increasing aging, aggregation, and microviscosity, which impair RBC deformability, cause capillary stasis, and reduce oxygen delivery (10, 11). High RBCs may enhance oxygen delivery to retinal tissues, while low RBCs or even anemia may worsen hypoxic conditions, contributing to the progression of DR (9, 12). Additionally, RBCs play a role in systemic inflammation crucial in diabetes-related complications (9). Dysfunctional RBCs in diabetes promote systemic inflammation by interacting with endothelial cells and releasing inflammatory mediators, worsening chronic inflammation and tissue damage (9, 14). This oxidative stress also reduces nitric oxide bioavailability and endothelial function, contributing to vascular dysfunction and accelerating the progression of DR (14). This underscores a dual role for RBCs: while they can potentially protect against DR through enhanced oxygen delivery, dysfunction or alterations in RBC properties may contribute to vascular damage. While our findings suggest an association between higher RBC counts and reduced DR risk, causality cannot be inferred due to the cross-sectional design. Future longitudinal studies are needed to confirm temporal relationships.

Our predictive model demonstrated that duration is an independent predictor of DR. Prolonged exposure to elevated blood sugar levels causes damage to the retinal blood vessels, triggering inflammation, oxidative stress, and the leakage of fluid from capillaries (15). As the duration of diabetes increases, these effects become more pronounced, leading to more severe stages of DR, such as proliferative DR, which can threaten vision (15, 16, 18, 19). Although good control of blood glucose levels can help reduce the risk of DR, individuals with long-term diabetes still face a higher likelihood of developing retinal damage.

This study found that DR is closely linked to DPN due to shared factors like high blood sugar, oxidative stress, and small blood vessel damage (16, 20). Both conditions are caused by microvascular problems, and DPN may signal vascular issues affecting the retina (16, 20). People with DPN are more likely to develop DR because both the nerves and retina undergo similar changes (21). DPN may indicate early retinal damage, emphasizing the need for regular eye exams for diabetic patients. Early treatment of DPN could help prevent or slow DR. Our results show a strong link between DPN and DR, with an odds ratio of 4.15.

The present study identified a correlation between SCR and DR in individuals with T2DM, corroborated by earlier studies (18, 19, 22). Research has shown that increased creatinine levels, which signal kidney impairment, often occur alongside diabetic retinopathy due to common vascular weaknesses affecting both the kidneys and the retina (22, 23). DKD is linked to a heightened risk of DR, as both systems experience damage due to sustained elevated blood glucose levels, resulting in endothelial injury, inflammation, and oxidative stress (23, 24). Monitoring creatinine levels and effective management of DKD can assist in identifying individuals at increased risk for DR. Given that DKD is a significant risk factor for DR and also strongly influences RBC/Hb levels through erythropoietin, we established Model A (primary) and Model B (excluding DKD). Our results showed no significant difference between the two models, suggesting that the confounding effect of DKD is relatively small(Attached Table).

In recent years, emphasis has been placed on developing predictive models for DR in patients with T2DM; however, few studies have examined blood cytological indicators, particularly RBC, resulting in the neglect of RBC’s effect on DR in models. A DR risk prediction nomogram was developed, identifying five key predictive variables, which exhibited robust performance with an AUC of 0.707 during internal validation, emphasizing its accuracy and discriminatory ability. Additionally, the model demonstrated excellent results in the Hosmer-Lemeshow test, calibration curves, and DCA, highlighting its potential utility in clinical DR risk assessment and management.

Our study offers guidance for healthcare professionals and patients on preventing DR. Early detection and management are crucial to avoid severe visual impairment. Regular monitoring of RBC count, renal function, and the progression of DPN and DKD is essential, particularly as diabetes duration increases. Healthcare providers should monitor these indicators through blood tests and clinical assessments. Patients should also be educated on maintaining optimal blood glucose control, managing body weight (BMI < 24 kg/m²), and engaging in regular aerobic exercise (2–3 sessions per week). Proactive monitoring and early intervention are key to slowing DR progression and improving quality of life for individuals with diabetes.

However, this study presents certain limitations. Firstly, the observed association between RBC count and DR may be affected by confounding variables such as blood pressure, inflammatory biomarkers or medications that may influence RBC count or DR risk. Future studies should incorporate these variables to refine risk estimates. Secondly, external validation in multiethnic cohorts is critical to confirm our findings. Collaborative efforts with institutions in diverse regions are planned for follow-up studies. Lastly, the wider confidence interval reflects both the imprecision of the estimation and the small sample size, and future studies will expand the sample size to improve accuracy.

## Conclusions

The findings suggest RBC levels may represent a biomarker associated with lower DR prevalence. A risk prediction model incorporating RBC count and other clinical factors was developed, which showed strong predictive value for DR risk. These findings indicate that RBC count could be instrumental in the early identification of high-risk diabetic patients, contributing to the prevention and management of DR. Further research should validate this association in prospective cohorts and examine potential biological pathways.

## Data Availability

The raw data supporting the conclusions of this article will be made available by the authors, without undue reservation.
