# Supplementary material for: Red blood cell count and its inverse association with diabetic retinopathy: Exploratory development of a risk assessment model in a retrospective cohort
Source: Front Endocrinol (Lausanne). 2025 Aug 8;16:1571192. doi: 10.3389/fendo.2025.1571192 (PMC12375921; doi:10.3389/fendo.2025.1571192)
Supplement: Supplementary file 1 [file Table1.doc]

Supplementary Table 1 Multivariate logistic regression analyses for patients with T2DM(Model A and Model B).

|  | Model A | | Model B | |
| --- | --- | --- | --- | --- |
| Variables | OR(95%CI) | P value | OR(95%CI) | P value |
| Duration(years) | 1.04(1.00-1.08) | 0.040 | 1.05(1.01-1.09) | 0.008 |
| RBC(×10^12/L) | 0.58(0.36-0.94) | 0.028 | 0.58(0.36-0.94) | 0.027 |
| SCR(umol/L) | 0.98(0.96-0.99) | 0.006 | 0.98(0.97-1.00) | 0.038 |
| DPN, n (%) | 4.15(2.20-7.83) | <0.001 | 3.99(2.15-7.43) | <0.001 |
| DKD, n (%) | 3.49(1.56-7.80) | 0.002 |  |  |
